# Delayed Emergency Department Diagnosis of Rat‐Bite Fever

**DOI:** 10.1155/crdi/8876337

**Published:** 2026-05-17

**Authors:** Christopher A. Anderson, Matthew P. Lazio

**Affiliations:** ^1^ Department of Emergency Medicine, SSM Health St. Agnes Hospital-Fond Du Lac, Fond Du Lac, Wisconsin, USA; ^2^ MEP Health, Madison, Wisconsin, USA; ^3^ Department of Family Medicine, Adjunct Clinical Professor of Medicine, University of Wisconsin-Madison School of Medicine and Public Health, Madison, Wisconsin, USA, wisc.edu

**Keywords:** case report, presumptive diagnosis, rat-bite fever, *Streptobacillus moniliformis*

## Abstract

Rat bite fever, caused by *Streptobacillus moniliformis* and *Spirillum minus*, is a zoonotic infection with increasing prevalence as more people keep rats as pets. It is a challenging diagnosis due to a broad range of symptomatology, and its causative organisms are difficult to culture. As such, it may be necessary to be diagnosed presumptively in the appropriate clinical context. This report details a case where diagnosis was presumptively made on a second emergency department presentation after no diagnosis was made on the first presentation. The patient in this case presented with fever, rash, and arthralgias, though presenting symptoms can vary from case to case. While treatable with penicillin or other antibiotics, it may lead to severe complications when diagnosis is delayed or failed. This patient was successfully treated with ceftriaxone followed by cephalexin (chosen due to a penicillin allergy) and made a complete recovery without complication. Taking a complete and thorough history is essential to ensuring that the diagnosis is made and that therapy is initiated in a timely manner.

## 1. Introduction

Rat‐bite fever is an infection characterized by a bite from a rat followed by fever, rigors, and arthralgias, often with other associated symptoms that can vary from presentation to presentation. It is a challenging diagnosis to make, as its symptoms are widely variable, and both causative organisms are difficult to culture. This often leads to delay in treatment or failure of diagnosis [1]. We present a presumptive case of rat‐bite fever that was diagnosed at a subsequent emergency department (ED) presentation after going undiagnosed at the initial visit.

## 2. Case Report

A 37‐year‐old male presented to the ED with a two‐day history of fever, chills, rigors, headache, nausea, and vomiting. He had no significant medical history at the time of presentation. He reported living in a suburban home in an area where ticks are endemic, though he had not noticed any recent tick bites.

During his initial ED presentation, his vital signs were significant for a fever of 39°C (102.2°F), mild tachycardia with a heart rate of 102 beats per minute, a respiratory rate of 16 breaths per minute, a blood pressure of 138/78 mm of mercury, and O_2_ saturation of 97% on room air. He reported mild neck discomfort but offered no neurologic complaints. He exhibited no nuchal rigidity, focal weakness, or ataxia on exam. He had no rash or other significant exam findings.

Initial ED laboratory values are presented in Table 1. He received parenteral doses of metoclopramide, diphenhydramine, and ketorolac as well as an oral dose of acetaminophen for treatment of his headache. His symptoms improved throughout his ED encounter; he defervesced to 99.9°F, and his heart rate improved to 76. He was able to tolerate oral intake and ambulate without difficulty following treatment and workup. The patient was discharged home with strict return precautions.

**TABLE 1:** Laboratory values from initial and subsequent emergency department visits.

	Initial visit	Subsequent visit	Normal range
White blood cell count	15,000 ↑	12,000 ↑	4200–10,500 per microliter
Hemoglobin	13.2 ↓	12.1 ↓	13.8–17.3 g per deciliter
Hematocrit	37.5 ↓	34.7 ↓	39.5%–51.0%
Platelet count	179,000	183,000	150,000–450,000 per microliter
Neutrophils	90.8 ↑	84.9 ↑	42.0%–75.0%
Lymphocytes	3.0 ↓	4.3 ↓	16.0%–44.0%
Monocytes	6.0	10.6	2.0%–12.5%
Eosinophils	0.1 ↓	0.0 ↓	0.3%–7.0%
Basophils	0.1	0.2	0.0%–1.5%
Sodium (Na)	134 ↓	133 ↓	135‐143 milliequivalents per liter
Potassium (K)	3.8	4.0	3.5–4.9 milliequivalents per liter
Chloride (Cl)	101	99 ↓	100‐109 milliequivalents per liter
Bicarbonate (CO_2_)	26	28	23‐31 milliequivalents per liter
Blood urea nitrogen (BUN)	18	17	6–21 mg per deciliter
Creatinine (Cr)	1.08	0.91	0.64–1.27 mg per deciliter
Glucose	155 ↑	123 ↑	70–99 mg per deciliter
Aspartate aminotransferase (AST)	n/a	34	10–38 units per liter
Alanine aminotransferase (ALT)	n/a	44	7–52 units per liter
Alkaline phosphatase (ALK)	n/a	74	30–99 units per liter
Bilirubin total	n/a	0.8	0.3–1.5 mg per deciliter
Protein	n/a	6.3	6.1–8.0 g per deciliter
Albumin	n/a	3.5 ↓	3.9–5.1 g per deciliter
Lactic acid	n/a	1.8	≤ 2.0 mmol per liter
Procalcitonin	n/a	6.99 ↑	< 0.07 ng per milliliter
C‐reactive protein (CRP)	n/a	21 ↑	< 0.10–1.00 mg per deciliter
Erythrocyte sedimentation rate (ESR)	n/a	21.30 ↑	0–10 mm per hour
Urinalysis (UA)	n/a	3–5 red blood cells 6–10 white blood cells ↑ 1+ protein ↑ no bacteria	0–2, 3–5 red blood cells per high power field 0–5 white blood cells per high power field no protein no bacteria

*Note:* Laboratory tests, values from initial emergency department visits, values from subsequent emergency department visits, and our hospital’s reference ranges are presented. The units of each value are included with the reference ranges. A ↑ indicates a value above the normal reference range, whereas a ↓ indicates a value below the normal reference range.

Two days following his initial presentation, he returned to the ED for evaluation of continued malaise, subjective fevers, persistent headache, bilateral lower abdominal pain, rash on the bilateral feet and lower legs, and atraumatic left ankle pain. He returned to the ED because he was advised to seek reevaluation following development of a rash.

**FIGURE 1:**
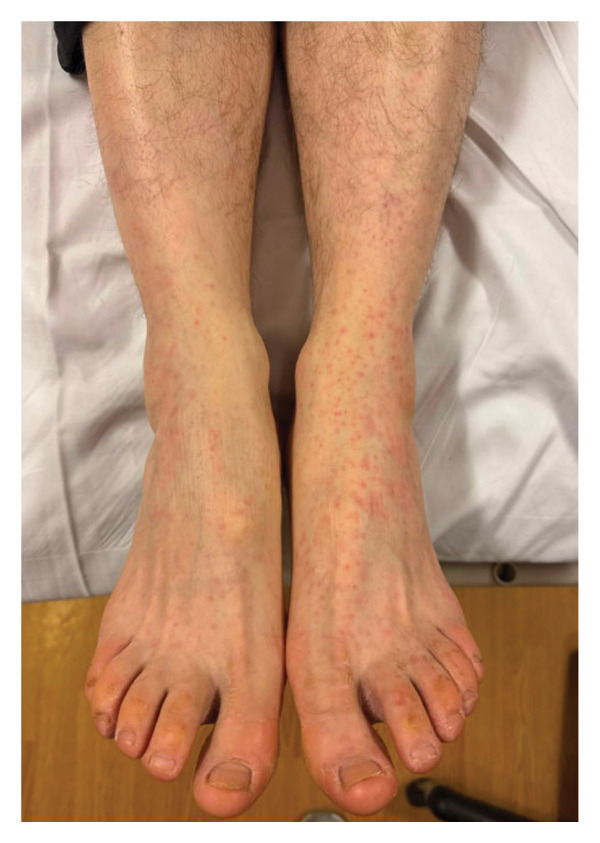
Petechial rash over distal bilateral lower extremities.

His vital signs at the second ED presentation were significant for a temperature of 36.8°C (98.3°F), heart rate of 55 beats per minute, respiratory rate of 16 breaths per minute, blood pressure of 121/78 mm of mercury, and O_2_ saturation of 98% on room air. He had mild diffuse abdominal tenderness without rebound or guarding. He exhibited a petechial rash to the bilateral ankles and feet without tenderness, warmth, or erythema (Figure 1). He reported discomfort with movement of the left ankle. He had palpable dorsalis pedis and posterior tibial pulses. He was alert and oriented, moving all extremities, and had no focal neurologic deficits.

His diagnostic workup was broadened at his second evaluation. Laboratory values from his second ED visit are presented in column 3 of Table 1. Two blood cultures were obtained prior to initiation of antibiotics. A CT angiogram of the abdomen/pelvis was obtained, given the lower abdominal pain and petechiae, out of concern for vasculitis. This showed mild gastric, pyloric, and duodenal wall thickening without evidence of perforation consistent with gastritis and duodenitis. There were no significant vascular findings.

**FIGURE 2:**
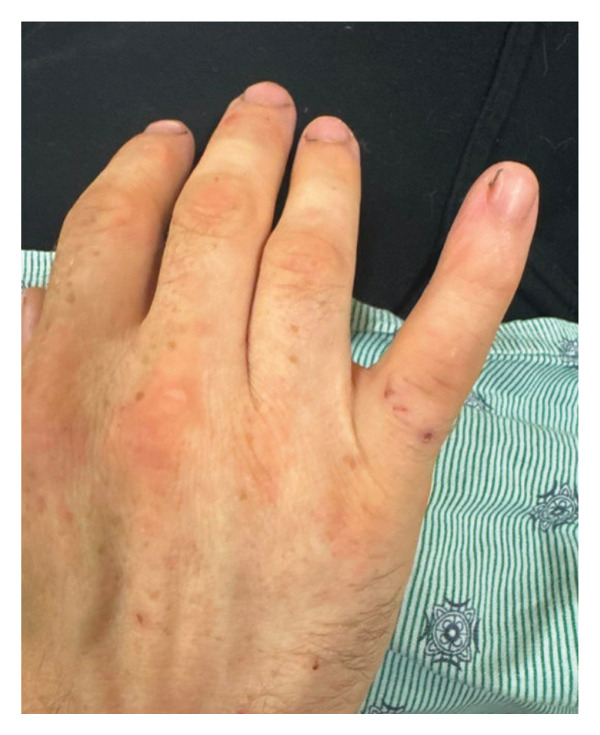
Paired puncture wounds overlying the right fifth metacarpophalangeal joint, representing the bite site.

While the workup was in progress during this second ED presentation, additional history was obtained in an effort to find a source of his infection. He denied tattoos, foreign travel, imprisonment, homelessness, and exposure to ticks and mosquitos. He is married in a monogamous relationship with his wife. He has two pet dogs and no cats. However, he also stated that he keeps two ball pythons as pets, and he buys feeder rats from a local pet store for their nutrition. He had recently bought a large quantity of rats and was keeping some of them alive for future feed preparation. He was bitten by one of the rats 4 days prior to his initial evaluation. Subsequent closer inspection showed two small puncture wounds on the right little finger (Figure 2). Personnel at the pet store in question identified these rats as members of *Rattus norvegicus*, a popular species of feeder rat in North America.

With this development in history, a working diagnosis of rat‐bite fever was made. He received a 2 g dose of ceftriaxone in the ED and was admitted to the hospital. He continued to receive ceftriaxone every 24 h throughout his hospital course and was discharged home on hospital day two following symptom improvement on 1000 mg cephalexin every 8 h for 10 days. This was likely dosed every 8 h rather than the more typical schedule of every 6 h in an effort to improve compliance. His blood cultures showed no growth after 14 days of incubation. He followed up with internal medicine after hospital discharge, at which time he reported doing well, and his physician documented that his infection had clinically resolved. He did not report any intolerance to antibiotics throughout his treatment course. The timeline of his care is summarized in the provided supplemental material.

## 3. Discussion

Two million people are bitten by animals every year in the United States, and rats account for around 1% of the total annual bites [2]. While rat‐bite fever is typically observed in young children living in poverty, a growing number of animals previously regarded as pests are being kept as pets. This exposes a greater proportion of the population to the risk of rat bites. The causative organism in the vast majority of reported cases in the United States is *Streptobacillus moniliformis*, though it is also caused by *Spirillum minus* in other parts of the world, predominantly in Asia [1]. The reported risk of infection following a bite is approximately 10% [3].

The differential diagnosis of a patient in the ED with fever, headache, rash, and arthralgias is broad. The patient was married in a monogamous relationship and reported no concern for sexually transmitted infection, so disseminated gonococcal disease or secondary syphilis was felt to be unlikely. He denied travel to areas endemic to *Rickettsia rickettsii*, so Rocky Mountain spotted fever was also considered unlikely. Although the patient lives in an area for which ticks are endemic, he had no significant time spent outdoors or known bites prior to symptom onset, so other tickborne infections were felt to be unlikely. He admitted to owning two dogs, but he had not sustained any bites or scratches from his dogs. He does not own cats, and as such infection by *Bartonella henselae* was felt to be unlikely. Meningitis was considered unlikely given the lack of toxicity and nuchal rigidity on exam. Encephalitis was also deemed unlikely due to his unremarkable neurologic exam and lack of altered mental status. Whereas Henoch–Schönlein purpura (IgA vasculitis) is predominantly a disease of childhood, it can affect adults and was considered. CT abdomen pelvis was performed to rule out an intraabdominal cause of his symptoms and showed findings of gastritis and duodenitis but was otherwise unremarkable.

Though blood cultures showed no growth, the diagnosis of rat‐bite fever remained the primary consideration. Our laboratory uses BACT/ALERT FA Plus and FN Plus bottles which are incubated and monitored using the VIRTUO system, typically for 4–5 days. In this case, the microbiology laboratory incubated the cultures for 14 days due to the requested extension by the treating clinical team. Despite modern blood culture practices, *S. moniliformis* is quite fastidious and can be inhibited by sodium polyanethol sulfonate, an anticoagulant, at concentrations that are typically used in blood culture media [3]. The difficulty in securing a microbiological diagnosis of rat‐bite fever, such as in this case, means that presumptive diagnosis may be necessary with the appropriate clinical findings and exposure history despite negative cultures.

This case demonstrates the importance of the collection of a thorough history during an ED presentation. Though consideration was given to a tick bite on initial presentation, the patient was not asked about other potential infection sources such as pets kept in the home. A detailed exam was conducted on each presentation, though the small size of the bite likely precluded detection without specific attention. Penicillin is typically chosen for treatment, though both doxycycline and cephalosporins have been used with success as well in penicillin‐allergic patients [4]. Timely diagnosis is essential, as untreated rat‐bite fever has had various described complications in literature including osteomyelitis and discitis as well as multiple cases of endocarditis [5, 6]. While the patient was ultimately discharged home from the hospital without complication after antibiotic initiation, a more rigorous history could have prevented a second ED workup and facilitated earlier treatment.

## 4. Conclusion

Although rare, the diagnosis of rat‐bite fever should be on the differential diagnosis for patients with fever, arthralgias, rash, and rat exposure. Treatment with penicillin or cephalosporins prevents complications such as osteomyelitis, discitis, and endocarditis.

## Funding

The authors received no funding support for the research, authorship, or publication of this case report.

## Disclosure

This manuscript has no available preprints and has not been presented at any scientific conferences or seminars.

## Consent

Written informed consent has been obtained from the patient for publication of this case report.

## Conflicts of Interest

The authors declare no conflicts of interest.

## Supporting Information

Additional supporting information can be found online in the Supporting Information section.

## Supporting information


**Supporting Information** A timeline of the patient’s illness and treatment is included as Supporting Information with this case report.

## Data Availability

The research involved in this case report has not generated any new data, and as such, the authors do not have any data available for sharing.
