# Supplementary material for: Delayed Emergency Department Diagnosis of Rat‐Bite Fever
Source: Case Rep Infect Dis. 2026 May 17;2026:8876337. doi: 10.1155/crdi/8876337 (PMC13181147; doi:10.1155/crdi/8876337)
Supplement: Supplementary file 1 — Supporting Information A timeline of the patient’s illness and treatment is included as Supporting Information with this case report. [file CRDI-2026-8876337-s001.docx]

The timeline of the patient’s care may be summarized as follows:

- Two days prior to symptom onset, he was bitten by a feeder rat
- He developed fever, chills with rigors, headache, nausea, and vomiting
- He initially presented to the ED on day two of symptoms and discharged home
- He returned to the ED on day four of symptoms, at which time he was diagnosed with rat-bite fever, initiated on ceftriaxone, and admitted to the hospital
- He was discharged home from the hospital on day six of symptoms, with a transition from ceftriaxone to oral cephalexin to facilitate home doses
- He followed up with internal medicine on day twelve of symptoms, and exhibited symptomatic and clinical resolution at that time
- He finished oral cephalexin course on day sixteen of symptoms
